# Assessment and Factors Contributing to the Quality of Life in Diabetes Mellitus Patients: A Cross-Sectional Single-Center Study

**DOI:** 10.7759/cureus.54359

**Published:** 2024-02-17

**Authors:** Abdullah Alaryni

**Affiliations:** 1 Internal Medicine, Imam Mohammad Ibn Saud Islamic University, Riyadh, SAU

**Keywords:** healthcare, saudi arabia, self-care behavior, quality of life, diabetes

## Abstract

Introduction

Diabetes mellitus (DM) is one of the most common chronic illnesses worldwide, with its prevalence rising rapidly every year. This condition adversely affects vasculature, leading to several potentially devastating complications like cerebrovascular mishaps, myocardial infarction, retinal damage, and renal compromise. These sequelae could lead to serious disabilities and negatively impact quality of life (QoL).

Objectives

This study aims to assess the QoL of adult diabetic patients in Saudi Arabia and determine influencing socio-demographic factors.

Methods

This is a cross-sectional study targeting adult diabetic patients visiting the Imam Medical Center in Riyadh, Saudi Arabia, from October 2022 to June 2023. The study encompasses diabetic patients of both genders aged 18 or more but excludes those diagnosed recently, i.e., within less than one year. A validated Arabic questionnaire, available online, was used to assess the QoL of the diabetic patients enrolled in this study. The sample size was 244 adult diabetic patients recruited from the Imam Medical Center through their official social media sites.

Results

The cohort of 244 patients recruited in this study were mostly aged between 18 and 30 (44.7%), females (52.5%), and married (47.5%). Furthermore, 58.6% of the participants were found to hold a bachelor's degree, 48.8% were employed, and 36.1% earned less than 5000 Saudi riyals each month. Of the total patients, 36.5% were diagnosed with diabetes 1-5 years ago, while 30.5% were diagnosed 5-10 years ago. Type 1 diabetes afflicted 48.4% of participants, whereas 47.1% were affected by type 2 diabetes. It was also determined that type 2 diabetic patients have higher overall QoL scores than type 1 diabetic patients. QoL had a high mean score of 22.05±4.4. The psychological/spiritual domain has the greatest mean score of 24.06±5.4, while the social and economic domain has the lowest (20.58±4.6). The majority of participants (71.3%) did not have other chronic conditions, whereas hypertension (18%) is the prevalent comorbidity, followed by respiratory ailments (7.4%) and cardiovascular disease (3.3%). Furthermore, people with hypertension had a higher total QoL and four life domain scores than those with cardiovascular and respiratory disorders.

Conclusion

The relevance of the findings is that it could aid health practitioners in developing techniques to encourage patients to undertake self-care to improve physiological management of the condition and reduce complications.

## Introduction

According to the World Health Organization, the prevalence of diabetes mellitus (DM) has quadrupled in less than four decades, from 1980 to 2014. This condition negatively affects vasculature, leading to potentially devastating complications like cerebrovascular accidents, myocardial infarction, retinal damage, renal compromise, and several others. These sequelae could lead to serious disabilities impacting the quality of life (QoL) of afflicted individuals [1]. Banegas et al. [2] investigated the cumulative impact of cardiovascular risk factors such as obesity, hypertension (HTN), and DM in a population comprising elderly individuals aged 60 years or more. The health-related QoL was considerably reduced in groups of those afflicted with only a singular cardiovascular disease (obesity, HTN, DM groups) or with more than one debilitating condition compared to those who did not suffer any of these conditions. Among men, DM was the most significant factor associated with the greatest decline in QoL [3]. Surprisingly, the QoL was less adversely affected in men suffering from all three conditions than expected. Obesity was identified as the most significant factor associated with the highest reduction in the QoL of women [3]. Groups with comorbid conditions showed a further reduction in QoL, with the lowest QoL observed in groups with obesity. Women suffering from all three conditions showed a higher reduction in QoL than was expected from summing up the reduction in QoL effectuated by each factor individually [3].

One question that should be asked is: Does DM affect life expectancy? Liang et al. conducted a study measuring the state of health in a population of elderly people afflicted with diabetes and HTN over four different periods [4]. Compared to men, the life expectancy of women has been found to be longer in groups suffering from DM or HTN [4]. However, despite this longer life expectancy, women's QoL tends to be lower [4]. Also, men suffering from HTN demonstrated a longer life expectancy but lower QoL, a trend comparable with their female counterparts [4]. In contrast, men with DM experienced a reduction in both life expectancy and QoL [4]. Another cross-sectional study evaluated the health-related QoL of 380 randomly selected DM patients in a tertiary clinic in Gaborone, Botswana [5]. The patients' socio-demographic and clinical factors were analyzed through a structured questionnaire, which revealed the adversely impacted mental and physical health and a relatively lower health-related QoL of Botswana's diabetic patients [5].

The cross-sectional study conducted by ALSharit and Alhalal in Al Ahsa, Saudi Arabia, showed that among the 256 participants, only 27.3% could comprehend and use health- and disease-related information to manage their condition and maintain glycemic control to prevent the complications that may arise from diabetes, while 38.3% of the population routinely needed help reading hospital reports and prescriptions from foot care to adherence to their medication [4].

Another cross-sectional study of 699 diabetic patients showed the association of age, gender, employment, and education level of a patient with their QoL, with young males having a high level of education and employment status experiencing the least burden of disease [6]. Foot ulcers are one of the complications that affect 2-3% of diabetic patients every year, causing 85% of leg amputations per year in the United States, while in the United Kingdom, 5.3% of diabetic patients have had foot ulcers at some point and 10.1 amputations per 100,000 citizens. It is also instrumental in determining a patient's QoL because a physician, when treating the condition, should take into consideration the cost and how it may affect the patient after that may not match the patient's thought of being well treated. The presence of this late complication may affect individual QoL from all aspects of their careers, physical, social, economic, and psychological, especially if diabetic nephropathy is present, which is why preventing foot ulcers is essential in improving the QoL through foot care with better technique; this will also lead to both faster detection and early intervention of foot ulcers which showed to improve the psychological aspect with limited effect on their career [7].

A further cross-sectional study, conducted at the Prince Sultan Military Medical City in Riyadh, Saudi Arabia, on 81 diabetic patients with foot ulcers assessed their health-related QoL and its related risk factors [8]. The participants were interviewed, and the Arabic version of the 36-Item Short Form Survey (SF-36) was administered to them to determine their health-related QoL. According to the research, the patients' physical activities were significantly affected by several socioeconomic factors like age, education, occupation, gender, and history of smoking, diabetes, dyslipidemia, HTN, body mass index (BMI), and related diabetes-associated complications [8]. The QoL pertaining to health was found to be lower in diabetic patients with foot ulcers in Saudi Arabia in this study [8].

Almasri et al. conducted a cross-sectional study at King Abdulaziz University Hospital in Jeddah, Saudi Arabia. The study employed the 5-level EQ-5D version (EQ-5D-5L) to assess the socio-demographic and clinical attributes along with the impact of DM on the QoL of 131 participants [7]. The results showed that in diabetic patients, several factors, such as gender, age, extent of physical activity, duration of affliction with diabetes, and comorbidities, could affect the QoL [7]. Therefore, assessing diabetic patients' QoL and determining the effect of various treatment modalities are indispensable for improving their health and well-being [9]. Another cross-sectional descriptive study conducted at the hospital's University Diabetic Center in 2016 assessed the interrelationship of attitude and knowledge with QoL pertaining to the health of 75 type 2 DM patients [10]. In type 2 diabetic patients, health-related QoL and knowledge scores were moderate [10]. The QoL in relation to health could be associated with a positive attitude toward the disease; most respondents believed in their responsibility for self-care and management [10].

A cross-sectional study was conducted between November 2017 and April 2018 at the Eastern Province's King Fahad Hospital in Saudi Arabia to study the QoL pertaining to the health of 378 patients afflicted with type 2 DM using the EuroQol Instrument and its predictors [11]. According to the study, a 0.808 median index score reflects moderate health-related QoL, with more than 25% of the studied population suffering from a debilitating health condition in some or all domains [11]. The results of multiple regression show that males with high monthly earnings, normal blood glucose levels (>200 mg/dl), and no history of diabetes or related complications will more likely attain a higher index score than alternative groups [11]. From November 2017 to May 2018, an observational cross-sectional study evaluated the QoL among 179 randomly selected diabetic patients between the ages of 30 and 60 years at Majmaah's primary healthcare facilities and hospital [12]. The survey revealed that diabetic patients had an average overall QoL [12]. This was also true for physical, social, and work functioning [12]. Current research outcomes, along with healthcare assessments of the QoL of diabetic people, fuelled a tremendous increase in the use of these evaluations as a technique for clinical research. The following are the objectives of diabetic patients' psychosocial well-being and QoL monitoring: identifying patients suffering from anxiety or depression, determining the psychological costs and benefits of new treatments, and determining why patients are unhappy with their care and management in general. This study addresses the satisfaction of diabetic patients in Saudi Arabia pertaining to different aspects of life, including health and function, social and economic status, psychological/spiritual contentment, and family values.

## Materials and methods

This cross-sectional questionnaire-based study was performed at the Imam Medical Center in Riyadh, Saudi Arabia, from October 2022 to June 2023. Both male and female diabetic patients with a confirmatory diagnosis from a registered physician and aged 18 or more were recruited. The study did not include patients younger than 18. Patients diagnosed within a year from the time of study were also excluded to ensure the recruitment of only those patients who had already corresponded with requisite experts like physicians, dietitians, educators, and social workers. During the study period, a total of 350 patients attended the healthcare facility, out of which 244 (70%) adult diabetic patients were enrolled.

An online validated Arabic questionnaire was employed as a tool to assess the QoL in diabetic patients enrolled in this study. The data was collected through the online self-administered questionnaire distributed as an online link through the Imam Medical Center's official social media platforms on Twitter (X) and WhatsApp. The responses received from the patients were further corroborated with their corresponding files in the hospital. The Institutional Review Board and the Ethical Committee at Imam Mohammad Ibn Saud Islamic University approved the study (approval number: 307/2022). Before the study's inception, the patients were briefed about the confidentiality statement, following which informed consent was obtained from the participants.

The questionnaire is composed of two parts as follows: (a) assessment of socio-demographic attributes including age, gender, education, social status, monthly income, employment status, presence of other chronic diseases, and the type of diabetes affecting the individual and when they were diagnosed (number of years since diagnosis) and (b) QoL assessment using a reliable Arabic version of the Ferrans and Powers Quality of Life Index (https://qli.uic.edu/). The QLI questionnaire instrument comprises two sections: the first evaluates a patient's satisfaction with a particular aspect of their life, and the second assesses the importance of that area of life for the patient. The responses, weighted by the importance ratings, indicate the patient's satisfaction with the aspect of life being appraised. Five scores are obtained for the Ferrans and Powers Quality of Life Index: (1) total QoL score, (2) health and functioning subscale score, (3) social and economic subscale score, (4) psychological/spiritual subscale score, and (5) family subscale score. Items listed below are from Part 1 (Satisfaction) and Part 2 (Importance).

The statistical analyses in this study were conducted using IBM SPSS Statistics for Windows, Version 23.0 (Released 2015; IBM Corp., Armonk, New York, United States) and GraphPad Prism version 8 for Windows (GraphPad Software, Boston, Massachusetts, United States, www.graphpad.com). A simple descriptive statistic was used to define the characteristics of the study variables through counts and percentages for the categorical and nominal variables. At the same time, mean and standard deviations present continuous variables.

## Results

Table 1 summarizes the participants' socio-demographic and clinical characteristics. In the cohort of 244 diabetic patients, 44.7% are aged between 18 and 30, 52.5% are females, and 47.5% are married. An assessment of the population's socioeconomic status revealed that 58.6% of the participants held a bachelor's degree, 48.8% of the patients were employed, and only 36.1% had a gross income lower than 5000 Saudi riyals. A majority of the patients were not afflicted with any other chronic conditions (71.3%), but the others had comorbidities such as HTN (18%), respiratory ailments (7.4%), and cardiovascular disease (3.3%). The prevalence of type 1 and type 2 diabetes was similar, afflicting 48.4% and 47.1% of the cohort, respectively. Confirmatory diagnosis was received by 36.5% of the population within 1-5 years of conducting this study, while 30.3% of the population received the diagnosis 5-10 years before this study.

**Table 1:** Participants' socio-demographic and clinical characteristics

Demographics		Count	%
Total		244	100
Age (years)	18-30	109	44.7
	31-50	84	34.4
	51-70	46	18.9
	>70	5	2
Gender	Male	116	47.5
	Female	128	52.5
Social status	Single	102	41.8
	Married	117	48
	Divorced	17	7
	Widowed	8	3.3
Education	Elementary school	4	1.6
	Middle school	4	1.6
	High school	64	26.2
	Bachelor's	143	58.6
	Masters/PhD	29	11.9
Employment status	Employed	119	48.8
	Unemployed	87	35.7
	Retired	36	14.8
	Disabled	2	0.8
Monthly income	<5000	88	36.1
	5000-10000	51	20.9
	10000-15000	58	23.8
	>15000	47	19.3
Were you diagnosed with any other chronic disease?	Cardiovascular disease	8	3.3
	Hypertension	44	18
	Respiratory diseases	18	7.4
	No	174	71.3
What type of diabetes mellitus?	Type 1 diabetes	118	48.4
	Type 2 diabetes	115	47.1
	Other	2	0.8
	Unknown	9	3.7
When were you diagnosed?	1-5 years	89	36.5
	5-10 years	74	30.3
	More than 10 years ago	81	33.2

This study investigated the participants' satisfaction in various aspects of life, including health and function, social and economic status, psychological/spiritual fulfillment, and family quality and values as per the Ferrans and Powers Quality of Life Index questionnaire. The reliability (internal consistency) of the variables determining the QoL score was appraised using Cronbach's alpha. Cronbach's alpha obtained from 34 items, as shown in Table 2, was 0.954, which indicates excellent internal consistency based on the rule of thumb provided by George and Mallery. Health and functioning and psychological/spiritual exhibit great internal consistency, with 0.918 and 0.905 alpha, respectively. Family variables had good internal consistency (0.897), while social and economic variables had acceptable internal consistency (0.744), as shown in Table 3.

**Table 2:** Cronbach's alpha obtained from 34 items

How satisfied are you with	1	2	3	4	5	6
Your health?	7 (2.9)	11 (4.5)	25 (10.3)	29 (11.9)	114 (46.9)	57 (23.5)
Your health care?	7 (2.9)	11 (4.5)	25 (10.3)	29 (11.9)	114 (46.9)	57 (23.5)
The amount of energy you have for everyday activities?	6 (2.5)	16 (6.6)	29 (11.9)	27 (11.1)	95 (38.9)	71 (29.1)
Your ability to take care of yourself without help?	1 (0.4)	8 (3.3)	14 (5.7)	29 (11.9)	82 (33.6)	110 (45.1)
Your ability to control your blood sugar?	11 (4.5)	16 (6.6)	21 (8.6)	68 (27.9)	85 (34.8)	43 (17.6)
The changes you have had to make in your life because of diabetes (such as diet, exercise, taking insulin or diabetes pills, checking blood sugar)?	1 (0.4)	13 (5.3)	21 (8.6)	47 (19.3)	87 (35.8)	74 (30.5)
The amount of control you have over your life?	9 (3.7)	9 (3.7)	18 (7.4)	51 (20.9)	88 (36.1)	69 (28.3)
Your chances of living as long as you would like?	4 (1.6)	6 (2.5)	16 (6.6)	24 (9.8)	85 (34.8)	109 (44.7)
Your family's health?	4 (2.8)	3 (2.1)	8 (5.6)	14 (9.9)	48 (33.8)	65 (45.8)
Your children?	4 (2.8)	3 (2.1)	8 (5.6)	14 (9.9)	48 (33.8)	65 (45.8)
Your family's happiness?	4 (2.8)	3 (2.1)	8 (5.6)	14 (9.9)	48 (33.8)	65 (45.8)
Your sex life?	5 (3.5)	7 (4.9)	8 (5.6)	21 (14.8)	48 (33.8)	53 (37.3)
Your spouse, lover, or partner?	5 (3.5)	7 (4.9)	8 (5.6)	21 (14.8)	48 (33.8)	53 (37.3)
Your friends?	5 (2.0)	5 (2.0)	13 (5.3)	25 (10.2)	73 (29.9)	123 (50.4)
The emotional support you get from your family?	5 (2.0)	5 (2.0)	14 (5.7)	23 (9.4)	69 (28.3)	128 (52.5)
The emotional support you get from people other than your family?	11 (4.5)	4 (1.6)	19 (7.8)	31 (12.7)	78 (32.0)	101 (41.4)
Your ability to take care of family responsibilities?	4 (1.6)	4 (1.6)	12 (4.9)	20 (8.2)	81 (33.2)	123 (50.4)
How useful you are to others?	2 (0.8)	4 (1.6)	9 (3.7)	26 (10.7)	85 (34.8)	118 (48.4)
The amount of worries in your life?	19 (7.8)	14 (5.7)	19 (7.8)	37 (15.2)	94 (38.5)	61 (25.0)
Your neighborhood?	6 (2.5)	9 (3.7)	15 (6.1)	23 (9.4)	85 (34.8)	106 (43.4)
Your home, apartment, or place where you live?	6 (2.5)	9 (3.7)	15 (6.1)	23 (9.4)	84 (34.4)	107 (43.9)
Your job (if employed)?	1 (0.8)	2 (1.7)	7 (5.9)	11 (9.2)	50 (42.0)	48 (40.3)
Not having a job (if unemployed, retired, or disabled)?	11 (8.8)	9 (7.2)	11 (8.8)	17 (13.6)	32 (25.6)	45 (36.0)
Your education?	9 (3.7)	3 (1.2)	13 (5.3)	27 (11.1)	91 (37.3)	101 (41.4)
How well you can take care of your financial needs?	8 (3.3)	11 (4.5)	24 (9.8)	39 (16.0)	71 (29.1)	91 (37.3)
The things you do for fun?	10 (4.1)	12 (4.9)	12 (4.9)	32 (13.1)	82 (33.6)	96 (39.3)
Your chances for a happy future?	5 (2.1)	5 (2.1)	9 (3.7)	23 (9.5)	80 (33.1)	120 (49.6)
Your peace of mind?	12 (4.9)	14 (5.7)	14 (5.7)	25 (10.2)	85 (34.8)	94 (38.5)
Your faith in God?	5 (2.0)	4 (1.6)	9 (3.7)	19 (7.8)	54 (22.1)	153 (62.7)
Your achievement of personal goals?	9 (3.7)	7 (2.9)	10 (4.1)	36 (14.8)	89 (36.5)	93 (38.1)
Your happiness in general?	6 (2.5)	6 (2.5)	21 (8.6)	27 (11.1)	73 (29.9)	111 (45.5)
Your life in general?	4 (1.6)	7 (2.9)	15 (6.1)	28 (11.5)	77 (31.6)	113 (46.3)
Your personal appearance?	5 (2.0)	5 (2.0)	11 (4.5)	22 (9.0)	76 (31.1)	125 (51.2)
Yourself in general?	4 (1.6)	7 (2.9)	15 (6.1)	28 (11.5)	77 (31.6)	113 (46.3)

**Table 3:** Social and economic variables QoL: quality of life

Reliability statistics	Cronbach's alpha	Number of items
Total QoL	0.954	34
Health and functioning	0.918	14
Social and economic	0.744	8
Psychological/spiritual	0.905	7
Family	0.897	5

The mean score for QoL and four domains is shown in Table 4. According to the findings, the psychological/spiritual domain has the greatest mean score of 24.06±5.4, while the social and economic domain has the lowest (20.58±4.6). QoL had a high mean score of 22.05±4.4.

**Table 4:** The mean score for QoL and four domains QoL: quality of life; SD: standard deviation

Domains	N	Min	Max	Mean	SD
Total QoL	244	4.15	29.56	22.05	4.4
Health and functioning	244	2.00	30.00	22.42	4.7
Social and economic	244	2.63	28.13	20.58	4.6
Psychological/spiritual	244	6.00	30.00	24.06	5.4
Family	244	2.40	30.00	20.96	5.6

The correlations between domains were determined using a two-tailed t-test at 0.01 significance level. Health and functioning are related to social and economic factors, as well as to psychological/spiritual and family (all p<0.001). The social and economic domain is also significantly related to psychological/spiritual and family factors. Furthermore, there is a significant relationship between psychological/spiritual and family factors (Table 5).

**Table 5:** Significant relationship between psychological/spiritual and family factors **: correlation is significant at the 0.01 level (two-tailed)

Correlations		Social and economic	Psychological/spiritual	Family
Health and functioning	r	0.777**	0.815**	0.644**
	p-value	<0.001	<0.001	<0.001
	N	244	244	244
Social and economic	r		0.692**	0.546**
	p-value		<0.001	<0.001
	N		244	244
Psychological/spiritual	r			0.577**
	p-value			<0.001
	N			244

Table 6 demonstrates factors strongly associated with QoL and the four domains. Age, social status, occupation, and monthly income were discovered to be significantly related to QoL and all four domains. Gender is associated with QoL and all domains except the psychological/spiritual category. Education, on the other hand, is strongly associated with the social and economic domains. Regarding clinical characteristics, having another chronic condition is significantly related to QoL and all dimensions, whereas having diabetes is significantly related to QoL except for the psychological/spiritual category. The 51-70 age group has a higher QoL score than the others. They also score higher on health, functioning, and family. Participants between the ages of 31 and 50, on the other hand, have greater social and economic scores than others. Males have a higher QoL than females. Married patients have higher overall QoLs and ratings on four social status areas. Participants with master's degrees had higher QoL and four domain ratings. Regarding employment, retired individuals scored higher in QoL, health and functioning, psychological/spiritual, and family domains, whereas employed participants scored higher in social and economic areas. Those earning more than 15000 Saudi riyals have a superior overall QoL in all aspects except psychological and spiritual.

**Table 6:** Factors associated with QoL and the four domains a: significant using one-way ANOVA test at <0.05 level; b: post hoc test=LSD; c: post hoc test=Games-Howell; d: significant using independent t-test at <0.05 level; e: significant using Welch's t-test at <0.05 level QoL: quality of life; ANOVA: analysis of variance; LSD: least significant difference

Demographics		Total	Total QoL	Health and functioning	Social and economic	Psychological/spiritual	Family
Age (years)	18-30	109	20.87±4.0^A^	21.53±4.6^A^	19.77±4.5^A^	23.44±5.5^A^	17.69±3.7^A^
	31-50	84	22.73±4.5^B^	22.93±4.4^B^	21.65±4.7^B^	23.83±5.7^A^	22.67±5.4^B^
	51-70	46	23.74±4.2^B^	23.87±4.8^B^	20.74±4.4^AB^	26.14±3.8^B^	25.36±5.0^C^
	>70	5	20.75±5.6A^B^	19.78±6.4A^B^	18.96±2.9^AB^	22.59±7.2^AB^	23.22±6.3^ABC^
p-value			0.001^a,b^	0.011^a,b^	0.033^a,b^	0.030^a,c^	<0.001^a,c^
Gender	Male	116	23.00±4.0	23.18±4.2	21.72±4.1	24.59±4.9	22.64±5.3
	Female	128	21.19±4.6	21.73±5.0	19.55±4.7	23.59±5.7	19.45±5.4
p-value			0.001^e^	0.015^e^	<0.001^d^	0.146	<0.001^d^
Social status	Single	102	20.92±3.7^A^	21.77±4.3^A^	19.88±4.5^A^	23.82±5.2^A^	16.74±1.5^A^
	Married	117	23.61±4.2^B^	23.57±4.6^B^	21.76±4.2^B^	25.18±4.6^A^	24.79±5.1^B^
	Divorced	17	19.94±5.4^AB^	20.20±4.8^AC^	18.47±5.3^A^	20.61±7.4^A^	20.98±5.9^BC^
	Widowed	8	18.06±4.9^AB^	18.49±5.8^C^	16.74±3.9^A^	18.19±6.7^A^	18.81±4.0^AC^
p-value			<0.001^a,c^	<0.001^a,b^	<0.001^a,b^	<0.001^a,c^	<0.001^a,c^
Education	Elementary school	4	18.82±4.2	17.88±5.3	17.39±2.9^AB^	21.27±5.4	20.23±7.3
	Middle school	4	19.79±6.6	19.76±8.3	17.13±4.9^AB^	22.36±7.5	21.25±8.5
	High school	64	21.73±4.7	21.95±5.1	19.64±4.4^B^	23.98±5.5	21.68±5.9
	Bachelor's	143	22.05±4.2	22.56±4.3	20.82±4.7^AB^	24.01±5.4	20.30±5.1
	Masters/PhD	29	23.50±4.2	23.73±4.6	22.43±3.6^A^	25.10±5.0	22.75±6.2
p-value			0.148	0.087	0.016^a,c^	0.633	0.185
Employment status	Employed	119	23.04±4.2^A^	23.07±4.5^A^	22.79±4.2^A^	24.46±5.1^A^	21.70±5.5^A^
	Unemployed	87	20.01±4.3^B^	20.96±5.0^B^	17.90±4.1^B^	22.55±5.9^A^	17.80±4.3^B^
	Retired	36	23.80±3.2^A^	23.94±3.8^A^	19.89±3.0^C^	26.59±3.3^B^	26.13±3.6^C^
	Disabled	2	20.01±4.9^AB^	19.75±3.6^AB^	18.25±4.5^ABC^	20.86±9.3^AB^	21.95±2.5^ABC^
p-value			<0.001^a,b^	0.001^a,b^	<0.001^a,b^	0.001^a,c^	<0.001^a,c^
Monthly income	<5000	88	20.00±4.5^A^	20.74±5.2^A^	18.13±4.4^A^	22.65±6.1^A^	17.73±4.4^A^
	5000-10000	51	21.18±4.6^A^	21.35±4.7^A^	19.67±4.7^A^	22.87±6.2^A^	21.23±5.7^B^
	10000-15000	58	24.05±3.3^B^	24.30±3.7^B^	22.72±3.9^B^	25.96±3.6^B^	23.22±4.9^B^
	>15000	47	24.35±2.8^B^	24.40±3.0^B^	23.53±2.3^B^	25.65±3.5^B^	23.95±5.1^B^
p-value			<0.001^a,c^	<0.001^a,c^	<0.001^a,c^	<0.001^a,c^	<0.001^a,c^

Furthermore, people with HTN had a higher total QoL and four life domain scores than those with cardiovascular and respiratory disorders. It was also discovered that type 2 diabetic patients have higher overall QoL scores than type 1 diabetic patients.

Using the general linear model (GLM) at <0.05 significance level, the analysis further found out that monthly income less than 5000 (p=0.041) and 5000-10000 (p=0.027), type 1 (p=0.042) and type 2 diabetes (p=0.049), and other chronic diseases such as cardiovascular diseases (0.001), HTN (p=0.002), and respiratory diseases (p<0.001) are factors significantly contributing to overall QoL scores. Factors such as a monthly income of 5000-10000 (p=0.049), type 1 diabetes (p=0.047), and having a history of other chronic conditions all contributed considerably to the score in the health and functioning category. Similarly, having a monthly income of less than 5000 (p=0.007) Saudi riyals and a monthly income of 5000-10000 Saudi riyals (p=0.039) and other chronic conditions are strongly associated with the social and economic domain. Age between 18 and 30 years old (p=0.030), being single (p=0.001) and married (p=0.002), having a monthly salary between 5000 and 10000 (p=0.040), and having other chronic diseases are all significant factors in the psychological/spiritual category. Being married (p=0.004) and having HTN (p=0.031) and respiratory disease (p=0.03) are significantly associated with the family domain.

## Discussion

Diabetes is a chronic condition with numerous major short- and long-term implications that impair the patient's QoL. Diabetic patients are expected to boost their QoL through self-management and long-term metabolism control. This study aimed to assess the impact of diabetes on the QoL of diabetic Saudi Arabians and to determine predictive factors associated with QoL.

The findings showed that diabetes affects QoL in many domains for diabetic patients. The highest and lowest scores were associated with psychological/spiritual and social and economic domain categories, respectively. At least three dimensions of QoL were significantly associated with age, gender, social status, employment, occupation, and diabetes-related comorbidities.

Demographic characteristics such as gender, age, education level, marital status, work status, and disease duration significantly affected all dimensions of patients' QoL in this study. Prior research conducted by Olukotun et al. [13] and AbuAlhommos et al. [14] determined distinct effects of demographic characteristics on the QoL of diabetic patients.

The QoL of diabetic males was perceived to be higher than that of diabetic female participants in this study. The result is similar to studies with other populations [6,15,16]. The consistency of these findings with previous research further corroborates the association of a lower QoL with adult diabetic females [17,18]. In the case of four domains, the findings agree with Nielsen et al.'s study, wherein males have fewer diabetes-related concerns [18]. It has also been reported that gender disparities in health-related QoL could be attributed to socio-cultural inequalities between men and women. With this, it is important to develop measures that could enhance the QoL of diabetic patients, particularly women [19]. The current study found an association between social status and QoL in people with diabetes, with married individuals scoring higher than single, divorced, and widowed. According to a Brazilian study, people who stayed married despite gaining much more weight are significantly less likely to acquire diabetes than their divorced counterparts [20]. The quality of marriage was found to affect health-related QoL and disease adaptation in a survey conducted by the American Diabetes Association to assess the impact of marital status on glucose control in insulin-treated diabetics. Furthermore, several studies have found that uncontrolled diabetes impacts people's everyday interactions and social life [21].

In terms of education, a higher level of education could be associated with a higher QoL in the present study. Patients with lower education qualifications had lower QoL scores, a finding comparable with several studies that have demonstrated improved health outcomes and higher QoL in patients with higher education [16,18]. Jansson et al. [22] found that college-educated diabetic patients were much less likely to have signs of mental disturbance that indicate the existence of a clinical condition. These studies indicate that people with higher education levels can better comprehend diabetes and have greater compliance and self-care, resulting in a lower risk of complications.

Comorbidities that significantly impacted the QoL of diabetic patients in this study are cardiovascular diseases, HTN, and respiratory diseases. It was found that patients with HTN have better QoL than those who have cardiovascular and respiratory diseases. In the study of Almasri et al. [7], comorbidities, including heart disease, contributed to poor QoL of diabetic patients. One study reported that having a cardiovascular disease could negatively affect the QoL and increase the risk of morbidity and mortality [23].

In the study, type 2 diabetic patients have higher QoL and four life domain scores than type 1 diabetics. This agrees with the study of Currie and colleagues, wherein SF-36 scores of the type 2 diabetic group are higher than the type 1 diabetic group. However, different findings were found in the study of Imayama et al. [24], in which no difference in health-related QoL scores was observed between the two groups. The only significant differences found are in the number of comorbidities, and the BMI of the patients significantly correlated with health-related QoL.

Limitations

This study explores the impact of diabetes on the QoL of diabetic patients in Saudi Arabia. This study has some limitations. First, the study's cross-sectional design hinders us from studying the causal relationships between socio-demographic characteristics and QoL of diabetic patients. Second, potential biases such as respondent ad selection bias could be present since this study used a self-reported questionnaire.

## Conclusions

The impact of DM on the QoL of diabetic patients is highly variable and is determined by multifarious factors. This study discovered that patients' socio-demographic variables considerably impacted their QoL. The patients' educational levels and competent knowledge of their diseased condition can justifiably be related to a higher QoL, given that they routinely follow the necessary measures for self-care and management. Gender inequalities are also prominent in diabetes management, as is evident from the longer life expectancy but lower QoL of diabetic females compared to their male counterparts with similar conditions. This study and previous research conducted on the Saudi Arabian population reflect the need to address the socioeconomic and gender inequities to enable better management of health and chronic diseases such as diabetes.

Other chronic disorders like comorbid cardiovascular and respiratory ailments may also worsen QoL. This study's findings of lower QoL in patients with comorbidities are also commensurate with previous findings. The relevance of the findings is that it could aid health practitioners in developing techniques to encourage patients to undertake self-care to improve the physiological management of the condition and reduce complications.
